# Using an ensemble of statistical metrics to quantify large sets of plant transcription factor binding sites

**DOI:** 10.1186/1746-4811-9-12

**Published:** 2013-04-11

**Authors:** Parsa Hosseini, Ivan Ovcharenko, Benjamin F Matthews

**Affiliations:** 1 Department of Bioinformatics and Computational Biology, George Mason University, Manassas, Virginia, USA; 2 Computational Biology Branch, National Center for Biotechnology Information, National Institutes of Health, Bethesda, Maryland, USA; 3 Soybean Genomics and Improvement Laboratory, United States Department of Agriculture, Beltsville, Maryland, USA

## Abstract

**Background:**

From initial seed germination through reproduction, plants continuously reprogram their transcriptional repertoire to facilitate growth and development. This dynamic is mediated by a diverse but inextricably-linked catalog of regulatory proteins called transcription factors (TFs). Statistically quantifying TF binding site (TFBS) abundance in promoters of differentially expressed genes can be used to identify binding site patterns in promoters that are closely related to stress-response. Output from today’s transcriptomic assays necessitates statistically-oriented software to handle large promoter-sequence sets in a computationally tractable fashion.

**Results:**

We present Marina, an open-source software for identifying over-represented TFBSs from amongst large sets of promoter sequences, using an ensemble of 7 statistical metrics and binding-site profiles. Through software comparison, we show that Marina can identify considerably more over-represented plant TFBSs compared to a popular software alternative.

**Conclusions:**

Marina was used to identify over-represented TFBSs in a two time-point RNA-Seq study exploring the transcriptomic interplay between soybean (*Glycine max*) and soybean rust (*Phakopsora pachyrhizi*). Marina identified numerous abundant TFBSs recognized by transcription factors that are associated with defense-response such as WRKY, HY5 and MYB2. Comparing results from Marina to that of a popular software alternative suggests that regardless of the number of promoter-sequences, Marina is able to identify significantly more over-represented TFBSs.

## Background

### Definitions and presumptions

We define a list of transcription factor binding sites (TFBSs), *t*_1_,*t*_2_,…,*t*_*N*_, where *t*_*i*_ is either a DNA motif, *m*_*i*_ or position weight matrix (PWM), *w*_*i*_. The former is a variable-length character-string from the four-nucleotide DNA alphabet, while the latter is a two-dimensional matrix of preset weights.

A group, *G*_*a*_, is a FASTA file populated with user-provided promoter sequences. Let *G*_*a*_,*G*_*a*+1_,…,*G*_*N*_ represent a list of *N* groups such that *N*≥2. We define a contingency matrix, *c*_*i*_ as a 2×2 matrix, used to model *t*_*i*_ over-representation across *G*_*a*_ and *G*_*a*+1_. A set of statistical metrics, *S*, quantify degree of *t*_*i*_ over-representation given *c*_*i*_.

### Transcription factors and binding site representation

Plants are constantly surrounded by stimulus, be-they deletorious pathogens or positive stimuli such as light and nutrients. In order for the plant to respond to these signals, plants must utilize regulatory proteins known as transcription factors (TFs) to facilitate transcriptional reprogramming in a dynamic, tissue-dependent manner. These proteins bind to enhancer or promoter *cis-*elements and facilitate the recruitment of RNA polymerase II. This combinatorial binding of TFs facilitates downstream execution of adaptative signals in the face of drought, herbivory, and high salinity. By quantifying binding–sites for these regulatory proteins, inherent transcriptional dynamics and magnitude of over-representation can be inferred.

TFs are classified into families by inherent DNA-binding signatures otherwise known as protein domains. In *Arabidopsis thaliana*, for instance, there are 64 known TF families [1], and it is not uncommon for different TF family members to exhibit relatively similar functionality. This redundancy is especially true when it comes to stress-response [2-4].

DNA motifs and PWMs are two models frequently used to define a TFBS. The former is a short *cis-*element region presumed to be a TFBS, while the latter models nucleotide propensities of a TFBS in the form of a matrix [5,6]. PWMs have been used across a broad spectrum of plant investigations such as identification of conserved exonic splice-site enhancers in *Arabidopsis thaliana*[7], prediction of potential seed-storage regulatory elements in mustards, grasses and legumes [8], and identification of novel regulatory elements in *Arabidopsis thaliana*[9]. With assays such as ChIP-ChIP and ChIP-Seq, novel regulatory elements can be identified and modeled as a PWM [10].

## Implementation

Marina is an operating-system independent GUI software tool built using the Java programming language.

This manuscript builds on the works of Chekmenev et. al [11], Loots et. al [12], and Kel et. al. [13], by implementing multiple statistical metrics to identify the maximum number of biologically-sound TFBSs, while accounting for cases when large promoter sets are provided.

To begin analysis with Marina, at least two FASTA files populated with user-provided promoter sequences are required. Each FASTA file is known as a group. A group, for instance, could represent promoter sequences of interest for a particular condition or time point.

**Figure 1:**
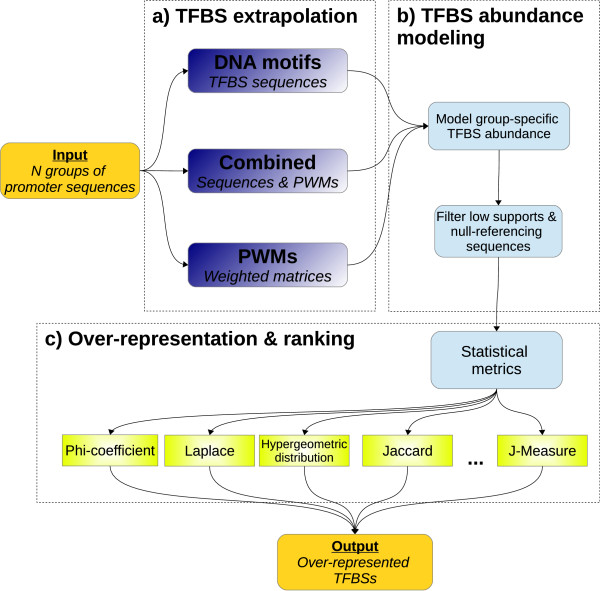
**Marina workflow. a)** A group is an umbrella-term to represent a set of promoter sequences. In order to run Marina, at least two groups must be provided. In doing so, TFBSs within each group can be contrasted and statistically quantified using TFBSs modeled as either DNA motifs or PWMs. Marina can also run if both these data-structures are provided, hence the name combined. **b)** Each group is modeled as a uni-directional graph, providing a means of trimming low-abundant promoter-sequences and TFBSs. **c)** A diverse collection of statistical metrics are used to model and quantify TFBS abundance. Magnitude of TFBS abundance is ranked and the hypergeometric distribution p-value computes significance of TFBS over-representation.

The Marina workflow (Figure 1) is partitioned into three distinct phases. The first phase performs abundance-estimation given a catalog of known TFBS models (Figure 1a). Initial abundance derivation is performed via mapping of the TFBS onto user-provided promoter sequences. Following TFBS mapping, low-quality TFBSs are removed (Figure 1b). Finally, a collection of statistical metrics quantify and rank TFBS over-representation (Figure 1c).

### Phase 1: Binding site mapping

In order to effectively quantify TFBS abundance using this tool, TFBS models must be provided. These models are in the form of either DNA motifs or PWMs. Cumulatively, 1,240 TFBS models were mined and utilized throughout this study. Of these models, 1,160 were DNA motifs with the remaining 80 being PWMs; motif-to-PWM ratio of 13:1.

Plant DNA motif and PWM models originated from AthaMap [14], AGRIS [15], PlantCARE [16], TRANSFAC [17], and JASPAR [18]. DNA motifs and PWMs were stored in either a tab-delimited or FASTA file format, respectively. Due to licensing restrictions, Marina does not come pre-packaged with a catalog of TFBS models, however several PWMs are provided, built from known PDB structures using the 3DTF web-server [19]. Be it PWMs or DNA motifs, a user-friendly schema is provided for importing custom TFBS profiles.

#### DNA motif and PWM mapping

To efficiently derive over-representation using DNA motifs, Marina scans promoter sequences for any occurrence of this motif using the Boyer-Moore-Horspool algorithm [20]. Due to the short length of many DNA motifs, elements such as ARF1 (TGTCTC) [21] may ubiquitously map throughout a promoter sequence with many mappings having little biological significance. Though this tool provides the option to filter short-length models be it PWMs or DNA motifs, resultant abundance estimations may seldom be biologically significant simply due to the likelihood of spurious mappings.

Marina maps each PWM onto promoter sequences using a concurrent implementation of the P-MATCH algorithm [11]. P-MATCH calculates a likelihood that a particular candidate promoter region contains a TFBS. By default, Marina uses a probability-cutoff of 0.80; any sub-sequence with a score greater than this cutoff is rendered a potential TFBS.

Alongside DNA motif and PWM extrapolations is a third pseudo-extrapolation known as combined mode. This mode simply performs the two former extrapolations back-to-back, merging their results into a singular data-structure. Combined mode capitalizes on the abundance of DNA motifs and probabilistic power of PWMs.

### Phase 2: Modeling TFBS over-representation

TFBS abundances across all promoter sequences are modeled using a group-specific acyclic graph. Each graph is organized such that group name is the root-node and each TFBS is a child leaf node. Every TFBS node references a list of promoter sequences containing this TFBS.

Per graph child node, two measures are used to model initial TFBS abundance: raw counts and support [22]. The former is simply defined as the number of promoter sequences which contain this particular TFBS. Raw counts are a useful, comparable metric if all groups have approximately the same number of promoter sequences. Unfortunately some groups may be larger than others, resulting in skewed and uncontrastable counts. To circumvent this possibility, the latter probabilistic measure, support, comes in helpful. Support, *P*(*t*_*i*_,*G*_*a*_), is a data-mining metric for representing abundance of a TFBS within a particular group [22]. A collection of statistical metrics continue where support leaves off, providing a means of deducing TFBS abundance.

Both raw-counts and support serve as viable metrics to initially model TFBS abundance, however there may be cases were a rift between the two measures can appear. For example, suppose a single TFBS mapped only once to a group. Due to such minimal mapping, raw-count will be small but support would be large. Both low-support and low-count thresholds exist so as to filter corresponding graph nodes. Such graph trimming ensures that high-support and/or high-count TFBS nodes remain as they are more likely of having correlations to a particular group [23]. A caveat with threshold cutoffs is that low-abundance TFBSs will get discarded.

### Phase 3: Deriving over-represented TFBSs using numerous statistical metrics

Given remaining TFBSs nodes, Marina aims to deduce magnitude of over-representation per TFBS, *t*_*i*_ by contrasting its abundance across groups *G*_*a*_ and *G*_*a*+1_. To facilitate this objective, a collection of 7 knowledge discovery metrics, *S*, are implemented (Table 1). Though a single metric can theoretically suffice, employing the entire set provides a means to appreciate unique features per measure and avoid individual bias. This table is by no means exhaustive as there are well over 20 frequently used metrics [24,25]. The metrics in this table were selected so that there exists a sound mixture of both well-studied association and correlation measures.

**Table 1:** Statistical metrics

**Metric**	**Equation**	**Output range**	**Reference**
Confidence (CF)	*max*(*P*(*G*_*a*_|*t*_*i*_),*P*(*t*_*i*_|*G*_*a*_))	0…1	[26]
Cosine (CO)	$\frac{P(t_{i},G_{a})}{\sqrt{P(t_{i})P(G_{a})}}$	$0\ldots \sqrt{P(t_{i},G_{a})}\ldots 1$	[27]
Jaccard (JAC)	$\frac{P(t_{i},G_{a})}{P(t_{i})+P(G_{a})-P(t_{i},G_{a})}$	0…1	[28]
Kappa coefficient (K)	$\frac{P(t_{i},G_{a})+P(\bar{t_{i},G_{a}})-P(t_{i})P(G_{a})-P(\bar{t_{i}})P(\bar{G_{a}}))}{1-P(t_{i})P(G_{a})-P(\bar{t_{i}})P(\bar{G_{a}})}$	−1…1	[29]
Laplace Correction (LP)	$\text{max}\left(\frac{\text{NP}(t_{i},G_{a})+1}{\text{NP}(t_{i})+2},\frac{\text{NP}(t_{i},G_{a})+1}{\text{NP}(G_{a})+2}\right)$	0…1	[30]
Lift (LI)	$\frac{P(t_{i},G_{a})}{P(t_{i})P(G_{a})}$	0…*∞*	[31]
Phi coefficient (PHI)	$\frac{P(t_{i},G_{a})-P(t_{i})P(G_{a})}{\sqrt{P(t_{i})P(G_{a})(1-P(t_{i}))(1-P(G_{a}))}}$	−1…1	[32]

Given a group, *G*_*a*_, and a TFBS, *t*_*i*_, magnitude of TFBS over-representation can be determined using a set of statistical metrics.

In order to utilize such measures, TFBS abundances must be modeled in a suitable data-structure. A contingency matrix, *c*_*i*_, is an ideal data-structure candidate as it models TFBS distributions throughout multiple, independent groups (Table 2). Each metric within *S* processes frequencies within a contingency matrix, *c*_*i*_, so as to quantitatively deduce over-representation of TFBS, *t*_*i*_. Certainly not all metrics deduce magnitude of TFBS over-representation the same, resulting in difficulties as to which TFBSs are unanimously most over-represented by all metrics. A solution to bringing uniform over-representation agreement across all metrics is to standardize contingency matrix counts using Iterative Proportional Fitting (IPF) [33].

**Table 2:** Contingency matrices model TFBS over-representation

	***G***_***a***_	$\bar{G_{a}}$	
*t*_*i*_	*c*_*i*_(0,0)	*c*_*i*_(1,0)	*n*(*t*_*i*_)
$\bar{t_{i}}$	*c*_*i*_(0,1)	*c*_*i*_(1,1)	$n(\bar{t_{i}})$
	*n*(*G*_*a*_)	$n(\bar{G_{a}})$	*N*

TFBS abundance within specific groups can be modeled as a two-dimensional contingency matrix, *c*_*i*_.

#### Iterative Proportional Fitting (IPF)

IPF is an algorithm for standardizing counts in a two-dimensional contingency matrix such that matrix row and column marginals are equal to one another (Table 3). Through such adjustment, inherent associations and correlations can be discovered [34]. By performing IPF-standardization, output for all 7 metrics become normalized so as to agree which TFBSs are the most over-represented.

**Table 3:** IPF-standardization yields equal marginals in a contingency matrix

	***G***_***a***_	$\bar{G_{a}}$	
*t*_*i*_	*x*	*N*/2−*x*	*N*/2
$\bar{t_{i}}$	*N*/2−*x*	*x*	*N*/2
	*N*/2	*N*/2	*N*

Identical counts within diagonal cells leads to marginal rows and columns being equal to one another. Table adapted from [35].

Equations 1 and 2 present an implementation of the IPF algorithm originally outlined by Tan et al. [35]. The former equation adjusts counts, *a*, such that they are equal on the diagonal axis. The latter equation then subtracts the remainder of the counts from that of the entire matrix sum, *N*.

(1) $${c_{i}}_{1,0}={c_{i}}_{0,0}=a=\frac{N\sqrt{{c_{i}}_{1,1}{c_{i}}_{0,0}}}{2\left(\sqrt{{c_{i}}_{1,1}{c_{i}}_{0,0}}+\sqrt{{c_{i}}_{1,0}{c_{i}}_{0,1}}\right)}$$

(2) $${c_{i}}_{0,1}={c_{i}}_{1,0}=\frac{N}{2}-a$$

## Results and discussion

### Case study: over-represented *Glycine max* TFBSs during a *Phakopsora pachyrhizi* time-course infection

To evaluate the functionality of this software tool, we utilized a two time-course RNA-Seq study that investigates soybean (*Glycine max*) transcriptional dynamics upon pathogenesis with soybean rust (SR; *Phakopsora pachyrhizi*). As outlined in our previous study, susceptible Williams 82 soybean leaves were inoculated with SR and assayed using RNA-Seq 10 days after infection (dai) [36]. An accompanying uninoculated control was also assayed to serve as a baseline condition. In both the control and 10 dai samples, a total of 5,940,995 70bp reads and 5,574,892 40bp reads were respectively sequenced using the Illumina platform (GenomeAnalyzer IIx). Sequenced reads were deposited in NCBI SRA under accessions SRX100854, SRX129967 and SRX100853, SRX129959, respectively.

Per run, quality assessment and control (QA/QC) entailed removal of low quality reads and trimming of low-quality 3’ ends should its quality score be less than 22. Reads were also discarded if they mapped at least once to either the human genome (Hg19) or the JCVI Microbial Resource [37]. Upon QA/QC completion, a total of 5,015,459 control reads and 5,420,745 10 dai reads passed filtering; quality-scores of 27 and 30, respectively. For each time point, reads were mapped with at-most 3 nucleotide mismatches onto the soybean transcriptome build (Glyma 1.0) using BWA [38]. Custom Python scripts inferred differential expression by deriving RPKM [39] and $\text{lo}g_{2}\left(\frac{\text{RPK}M_{10\text{dai}}}{\text{RPK}M_{0\text{dai}}}\right)$ per transcript.

Two gene-sets were then declared to contain the top 600 induced and 600 suppressed differentially expressed genes (DEGs), respectively. Per gene set, the promoter sequence 2.5kb upstream from each genes transcription start site (TSS) was retrieved and appended to a FASTA file. Both FASTA files in-conjunction with 80 plant PWMs and 1,160 plant-specific DNA motifs served as input into Marina.

Marina identified 71 potentially over-represented TFBSs between the control and 10 dai groups (Table 4). As shown in this table, there exists no consensus amongst the various metrics as to which TFBS is truly the most over-represented. There are however some TFBSs that are ranked by all metrics in a relatively uniform manner: AG, ATHB6, and ABFS. For all other TFBSs, it is difficult to deduce magnitude of over-representation. Such a scenario warrants IPF-standardization as it normalizes metric-ranks to agree in-concert which TFBSs are the most over-represented (Table 5). By visually contrasting this table with that of Table 4, it is clear that unstandardized ranks from Laplace Correction (LP), Confidence (CF) and Lift (LI) perfectly equal their IPF-standardized counterpart.

**Table 4:** Various metrics infer differing magnitudes of TFBS over-representation

	**Metrics**								**TFBS raw-abundance**	
**TF**	**LP**	**CO**	**JAC**	**LI**	**CF**	**K**	**PHI**	**p-value**	**Suppressed**	**Induced**
ABF1	20	39	39	20	20	3	2	8.211e-274	130	169
ABFS	9	9	10	9	9	16	12	2.385e-31	10	20
ABI3/FUS3	67	19	17	67	67	41	58	3.036e-47	14	7
ABI4(2)	64	34	33	64	64	64	67	4.465e-172	66	43
AG	14	20	21	14	14	13	18	4.611e-82	30	42
AGP1	48	57	56	48	48	58	49	2.412e-720	427	398
ALFIN1	34	58	57	34	34	34	34	1.580e-731	440	426
ARF1	65	29	24	65	65	57	62	1.243e-113	40	25
ARR10	39	65	65	39	39	43	39	1.836e-895	579	552
ARR2	69	27	22	69	69	60	69	4.028e-99	33	15
ATHB-5	43	68	68	43	43	49	43	1.542e-901	584	555
ATHB1	40	67	67	40	40	45	40	3.162e-901	584	556
ATHB5-1	63	21	20	63	63	44	55	3.202e-78	26	18
ATHB5-2	37	60	60	37	37	37	37	9.771e-769	470	452
ATHB6	27	23	25	27	27	29	32	3.067e-109	41	46
ATHB9	53	38	36	53	53	55	52	2.105e-225	95	81
AtLEC2	55	51	51	55	55	68	61	1.066e-611	336	284
ATML1/PDF2	71	18	11	71	71	54	71	8.730e-38	10	1
AtMYB2	29	33	34	29	29	23	31	1.606e-170	70	76
AtMYB77	60	32	31	60	60	56	57	2.955e-141	53	40
AtMYC2	2	2	2	2	2	30	8	0.0002735	1	7
AtSPL3	30	45	46	30	30	8	26	7.997e-426	220	236
BLR/RPL/PNY	35	61	61	35	35	35	35	1.444e-777	478	462
bZIP910(2)	10	12	16	10	10	14	11	6.060e-42	14	26
bZIP911	12	11	13	12	12	19	14	4.350e-37	12	21
bZIP911(1)	11	10	12	11	11	20	13	2.529e-34	11	20
bZIP911(2)	18	13	14	16	16	32	29	3.730e-38	12	16
CBF	43	68	68	43	43	49	43	1.542e-901	584	555
CDC5	4	4	4	4	4	18	3	1.343e-10	3	13
DOF2	42	71	71	42	42	48	42	1.259e-902	585	556
DPBF1/2	51	55	55	51	51	66	54	1.857e-712	418	379
E2Fa	70	13	9	70	70	38	64	8.059e-24	6	1
E2Fc/d	1	1	1	1	1	26	5	0.0003077	1	8
EmBP-1	25	43	43	25	25	5	17	3.316e-397	203	228
GAMYB	47	59	59	47	47	53	47	2.040e-743	447	422
Gamyb	58	28	26	58	58	40	50	6.185e-120	44	36
GATA-1	17	24	28	18	18	12	16	5.923e-120	47	62
GATA-1/2/3/4	16	15	18	17	17	28	27	9.291e-54	18	24
GT-3b	13	25	29	13	13	7	7	1.244e-128	52	76
HAHB4	46	64	64	46	46	52	46	1.038e-891	575	546
HAT5	43	68	68	43	43	49	43	1.542e-901	584	555
HSE	19	26	30	19	19	11	15	1.641e-130	52	68
HVH21	41	66	66	41	41	46	41	3.881e-900	583	555
HY5	6	8	8	6	6	21	10	6.247e-20	6	15
ID1	28	31	32	28	28	27	33	4.015e-146	58	63
MYB.PH3(1)	56	41	41	56	56	61	56	4.159e-333	154	130
MYB.PH3(2)	52	49	49	52	52	62	53	6.937e-564	306	276
MYB98	62	36	35	62	62	65	65	2.648e-210	85	60
O2	33	56	58	33	33	6	28	2.967e-731	446	457
OsbHLH66	26	40	40	26	26	9	20	1.723e-308	147	165
OsCBT	3	3	3	3	3	24	6	1.543e-7	2	10
P	57	52	52	57	57	71	66	2.571e-629	347	286
PCF2	61	47	44	61	61	70	70	3.566e-441	215	160
PCF5	59	48	48	59	59	69	68	2.612e-498	254	201
PEND	31	35	37	31	31	15	30	3.825e-230	101	108
PIF3(2)	21	22	23	21	21	17	25	4.178e-99	37	46
RAP2.2	66	30	27	66	66	59	63	1.614e-125	45	28
RAV1(1)	49	54	53	49	49	63	51	1.957e-688	400	366
RAV1(2)	38	62	62	38	38	39	38	1.073e-854	543	519
STF1	24	37	38	24	24	10	22	1.243e-242	109	124
TAC1	68	17	15	68	68	42	59	1.479e-44	13	6
TaMYB80	54	50	50	54	54	67	60	2.700e-594	324	276
TBP	36	63	63	36	36	36	36	1.545e-881	568	547
TEIL	50	42	42	50	50	47	48	8.458e-340	160	146
TGA1	23	46	47	23	23	2	9	3.416e-468	253	293
TGA1a	32	53	54	32	32	4	23	3.325e-688	413	433
WRKY11	7	7	7	8	8	31	19	2.346e-14	4	9
WRKY18/40/62	7	6	6	7	7	33	21	2.879e-11	3	7
WRKY26/38/43	15	16	19	15	15	25	24	4.164e-56	19	26
WRKY6	5	5	5	5	5	22	4	1.091e-10	3	12
ZAP1	22	44	45	22	22	1	1	2.468e-415	219	268

Promoter sequences from the top 600 induced and top 600 suppressed genes 10 dai were identified and their TFBS abundances quantified using Marina. A catalog of pre-assembled DNA motifs (1,160 motifs) and PWMs (80 matrices) accompanied such groups.

A total of 71 over-represented TFBSs were identified. Of these *N* TFBSs, magnitude of over-representation is ranked from 1 to *N* such that the most over-represented are close to 1 while the least over-represented are close to *N*. Since TFBS models can vary across source-organisms, certain over-represented TFBSs were found multiple times (i.e. GAMYB, bZIP911, and ATHB5). Furthermore, not all metrics rank the same. As a result, manually deducing degree of TFBS over-representation can be a challenging task. IPF-standardization is designed to circumvent such a scenario.

**Table 5:** IPF-standardized abundances provides agreement amongst all metrics

	**Metrics**						
**TF**	**LP**	**CO**	**JAC**	**LI**	**CF**	**K**	**PHI**
ABF1	20	20	20	20	20	20	20
ABFS	9	9	9	9	9	9	9
ABI3/FUS3	67	67	67	67	67	67	67
ABI4(2)	64	64	64	64	64	64	64
AG	14	14	14	14	14	14	14
AGP1	48	48	48	48	48	48	48
ALFIN1	34	34	34	34	34	34	34
ARF1	65	65	65	65	65	65	65
ARR10	39	39	39	39	39	39	39
ARR2	69	69	69	69	69	69	69
ATHB-5	43	43	43	43	43	43	43
ATHB1	40	40	40	40	40	40	40
ATHB5-1	63	63	63	63	63	63	63
ATHB5-2	37	37	37	37	37	37	37
ATHB6	27	27	27	27	27	27	27
ATHB9	53	53	53	53	53	53	53
AtLEC2	56	56	56	56	56	56	56
ATML1/PDF2	71	71	71	71	71	71	71
AtMYB2	29	29	29	29	29	29	29
AtMYB77	60	60	60	60	60	60	60
AtMYC2	2	2	2	2	2	2	2
AtSPL3	30	30	30	30	30	30	30
BLR/RPL/PNY	35	35	35	35	35	35	35
bZIP910(2)	10	10	10	10	10	10	10
bZIP911	12	12	12	12	12	12	12
bZIP911(1)	11	11	11	11	11	11	11
bZIP911(2)	17	17	17	17	17	17	17
CBF	43	43	43	43	43	43	43
CDC5	4	4	4	4	4	4	4
DOF2	42	42	42	42	42	42	42
DPBF1/2	51	51	51	51	51	51	51
E2Fa	70	70	70	70	70	70	70
E2Fc/d	1	1	1	1	1	1	1
EmBP-1	25	25	25	25	25	25	25
GAMYB	47	47	47	47	47	47	47
Gamyb	58	58	58	58	58	58	58
GATA-1	18	18	18	18	18	18	18
GATA-1/2/3/4	16	16	16	16	16	16	16
GT-3b	13	13	13	13	13	13	13
HAHB4	46	46	46	46	46	46	46
HAT5	43	43	43	43	43	43	43
HSE	19	19	19	19	19	19	19
HVH21	41	41	41	41	41	41	41
HY5	6	6	6	6	6	6	6
ID1	28	28	28	28	28	28	28
MYB.PH3(1)	55	55	55	55	55	55	55
MYB.PH3(2)	52	52	52	52	52	52	52
MYB98	62	62	62	62	62	62	62
O2	33	33	33	33	33	33	33
OsbHLH66	26	26	26	26	26	26	26
OsCBT	3	3	3	3	3	3	3
P	57	57	57	57	57	57	57
PCF2	61	61	61	61	61	61	61
PCF5	59	59	59	59	59	59	59
PEND	31	31	31	31	31	31	31
PIF3(2)	21	21	21	21	21	21	21
RAP2.2	66	66	66	66	66	66	66
RAV1(1)	49	49	49	49	49	49	49
RAV1(2)	38	38	38	38	38	38	38
STF1	24	24	24	24	24	24	24
TAC1	68	68	68	68	68	68	68
TaMYB80	54	54	54	54	54	54	54
TBP	36	36	36	36	36	36	36
TEIL	50	50	50	50	50	50	50
TGA1	23	23	23	23	23	23	23
TGA1a	32	32	32	32	32	32	32
WRKY11	8	8	8	8	8	8	8
WRKY18/40/62	7	7	7	7	7	7	7
WRKY26/38/43	15	15	15	15	15	15	15
WRKY6	5	5	5	5	5	5	5
ZAP1	22	22	22	22	22	22	22

By having all metrics agree as to magnitude of over-representation per TFBS, the investigator will have an easier time identifying TFBSs of interest. Much like unstandardized ranks (Table 4), standardized ranks also range from 1 to *N* such that ranks in the vicinity of 1 are most over-represented while ranks in the vicinity of *N* are least over-represented.

#### Many over-represented TFBSs have defense or stress-response functions

Given the list of IPF-standardized TFBSs (Table 5), all 4 WRKY genes were over-represented at 10 dai. These abundances are supported by numerous studies which show that WRKY genes are perceived upon PAMP signals or abiotic stressors [40-43]. WRKY genes drive defense-response by regulating NONEXPRESSOR OF PR1 (NPR1) expression by binding to W-box motifs in the NPR1 promoter. NPR1 protein binds with TGA TFs which regulate pathogenesis-response (PR) expression, hence providing a means of positively regulating SA-defense response [44-46].

Similar to WRKY, a bZIP family TFBS, HY5, was also over-represented 10 dai. Inextricably linked to photomorphogenesis, this TF is also known for its positive regulation of auxin signalling; a phytohormone which regulates defense response [47,48]. Through interactions with HY1 and MYC2, HY5 is able to regulate photomorphogenesis, ABA and JA signaling [49,50].

Much like MYC2, AtMYB2 is not only over-represented at 10 dai but also plays a role in ABA-signaling. Microarray analyses on Arabidopsis plants with 35S:AtMYC2/AtMYB2 over-expression constructs revealed induced expression of several ABA-regulated genes [51].

The GT (Trihelix) TF family member, GT-3b, was over-represented at 10 dai. Much is unknown about this TF family let alone GT-3b, however what is known is that many GT members, like HY5, regulate photomorphogenic signaling [52]. A recent study showed how GT-2a and GT-2b over-expression positively-regulates ABA-signaling [53]. Though an over-expressed GT-3b construct was not part of this recent study, translating findings from GT-2a and GT-2b over to GT-3b could reveal potentially novel insights into whether GT-3b plays a part in ABA and defense-signaling roles.

#### Strong relationship between degree of TFBS over-representation and IPF-rank

**Figure 2:**
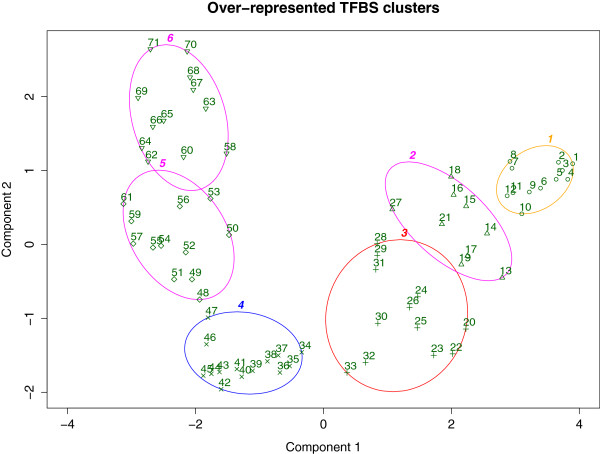
**Clustering of over-represented TFBS.** Performing dimensionality reduction on unstandardized TFBS ranks (Table 4) reveals distinct clusters of over-representative TFBSs. Each point in this 2-D coordinate plane references a unique TFBS, labeled based on its IPF-rank. From these 6 clusters, there appears to be a strong relationship between magnitude of TFBS over-representation and TFBS IPF-rank. The first two clusters, for instance, encapsulate all WRKY genes, GT-3b and HY5: genes perceived during defense response. This suggests that IPF-standardized ranks can elucidate magnitude of TFBS over-representation.

Due to the multi-dimensional nature of unstandardized TFBS ranks (Table 4), dimensionality reduction was performed to facilitate rank visualization on a 2D coordinate plane. To carry-out such analysis, Principle Component Analysis (PCA) followed by bi-variate clustering was executed using the R library clusplot [54]. All 71 TFBSs were partitioned into 6 discrete clusters and labeled based on their respective IPF-standardized rank (Figure 2). Interestingly, there appears to be a strong relationship between the magnitude of TFBS over-representation and IPF-standardized rank. This suggests that IPF-standardization is suitable for deducing magnitude of over-represented TFBSs.

#### Comparative software analysis

Several actively-used software tools and web-interfaces are available to quantify TFBS over-representation [14,15,18,55-57]. We classified such tools into two classes: software that deduce TFBS over-representation given either 1) one promoter-sequence set or 2) at least two promoter-sequence sets. Marina falls into this latter class and as does a popular software tool, F-MATCH [13]. Both tools require two FASTA files as input such that one file serves as a query sequence-set while the other a baseline control. Degree of over-representation is therefore deduced by statistically contrasting TFBS over-representation across these two groups.

Both software tools were compared using three independent sets of promoter-sequences of varying sizes. Each of these three analyses encompassed promoter-sequences of DEGs 10 dai from our prior soybean – soybean rust RNA-Seq study [36]. F-MATCH and Marina identify relatively the same number of over-represented TFBSs when promoter-sequence sets are 600 sequences in size (Table 6). As these promoter sets increase in size, Marina maintains steady consistency as to identification of over-representated TFBSs, while F-MATCH failed to detect any over-represented TFBSs. We believe the reasoning behind why F-MATCH yields 0 over-represented TFBSs while Marina identified almost 50 TFBSs to be attributed towards usage of the binomial distribution by F-MATCH, which is known to be sensitive to large test sets. As far as output consistency between the two tools, our only comparison pertains to results obtained with 600 sequences sets. Given the 44 F-MATCH and 47 Marina over-represented TFBSs, 21 TFBSs were shared between the two result-sets. Unlike F-MATCH, we did not include TRANSFAC Professional PWMs in our analysis. We believe by introducing such PWMs, the number of shared TFBSs would certainly increase.

**Table 6:** Comparing Marina and F-MATCH given catalogs of PWMs and DNA motifs

**Group size**	**PWMs (x 80)**		**DNA motifs (x 1,160)**	
**(# sequences)**	**F-MATCH**	**Marina**	**F-MATCH**	**Marina**
600	44	47	N/A	24
1500	0	50	N/A	41
2500	0	53	N/A	44

A collection of 80 plant-specific PWMs were supplied to Marina. When group-sizes are relatively small and PWMs are used, both Marina and F-MATCH identify approximately the same number of over-represented TFBSs. However as group-sizes increased, Marina consistently identifies over-represented TFBSs. Marina also accepts DNA motifs if PWMs are not available; F-MATCH does not accept such models.

## Conclusions

Marina is a operating-system independent software tool to identify over-represented TFBSs across different groups of promoter sequences. We illustrate its usage using an RNA-Seq plant-pathogen study, however promoter sequences from any organism can be analyzed using Marina as long as compatible TFBS models are provided. We also show its capability to identify over-represented TFBSs regardless of input size. Given large sets of DEGs, our results show that by contrasting their promoter sequences, TFBSs perceived during defense and stress response were significantly over-represented. Other lesser-known TFBSs joined these ranks, raising questions as to potential candidate TFs affiliated with defense-response.

The underlying algorithms within this tool are guided by a catalog of user-provided TFBS models be-it DNA motifs or PWMs. Thankfully, many regulatory element resources and databases exist. By contrasting this software tool to a popular alternative, we show that Marina is built for large promoter-sequence sets while being able to identify biologically sound over-representative TFBSs.

## Availability and requirements

**Project name:**Marina.

**Project home page:**http://mason.gmu.edu/~phossein/marina/

**Operating system(s):** Operating-system independent.

**Programming language:** Java version 7+.

**Other requirements:** None.

**License:** BSD.

## Abbreviations

ABA: Abscisic acid; CO: Cosine metric; CF: Confidence metric; DEG: Differentially expressed gene; IPF: Iterative proportional fitting; JA: Jasmonic acid; JAC: Jaccard; K: Cohen’s kappa; LP: Laplace correction; LI: Lift; NPR1: Non-expressor of PR1; PHI: Phi-coefficient; PWM: Position weight matrix; SR: Soybean rust; TF: Transcription factor.

## Competing interests

The authors declare that they have no competing interests.

## Authors’ contributions

BFM inspired development of Marina. PH wrote the manuscript, implemented the Marina software tool and underlying algorithms. IO guided the development of PWM extrapolation and over-representation analyses. All authors read, critiqued and approved the final manuscript.
